# Drone-Person Tracking in Uniform Appearance Crowd: A New Dataset

**DOI:** 10.1038/s41597-023-02810-y

**Published:** 2024-01-02

**Authors:** Mohamad Alansari, Oussama Abdul Hay, Sara Alansari, Sajid Javed, Abdulhadi Shoufan, Yahya Zweiri, Naoufel Werghi

**Affiliations:** 1 Department of Computer Science, Khalifa University, Abu Dhabi, UAE; 2 Department of Aerospace Engineering, Khalifa University, Abu Dhabi, UAE; 3 Advanced Research and Innovation Center (ARIC), Khalifa University, Abu Dhabi, UAE; 4 Khalifa University Center for Autonomous Robotic Systems (KUCARS), Khalifa University, Abu Dhabi, UAE; 5 Center for Cyber-Physical Systems (C2PS), Khalifa University, Abu Dhabi, UAE

**Keywords:** Computer science, Computational science

## Abstract

Drone-person tracking in uniform appearance crowds poses unique challenges due to the difficulty in distinguishing individuals with similar attire and multi-scale variations. To address this issue and facilitate the development of effective tracking algorithms, we present a novel dataset named D-PTUAC (Drone-Person Tracking in Uniform Appearance Crowd). The dataset comprises 138 sequences comprising over 121 K frames, each manually annotated with bounding boxes and attributes. During dataset creation, we carefully consider 18 challenging attributes encompassing a wide range of viewpoints and scene complexities. These attributes are annotated to facilitate the analysis of performance based on specific attributes. Extensive experiments are conducted using 44 state-of-the-art (SOTA) trackers, and the performance gap between the visual object trackers on existing benchmarks compared to our proposed dataset demonstrate the need for a dedicated end-to-end aerial visual object tracker that accounts the inherent properties of aerial environment.

## Background & Summary

The advancement of Unmanned Aerial Vehicles (UAVs), commonly known as drones, has significantly improved security surveillance capabilities. Drones excel at tracking and pinpointing individuals of interest, rendering person-following and tracking systems^1^ invaluable in domains like surveillance^2^, search and rescue missions^3^, and healthcare^4,5^. These systems leverage Visual Object Tracking (VOT) techniques, which involve locating and estimating the trajectory of specific objects within a sequence of consecutive frames^6,7^. VOT finds applications in various fields, including autonomous vehicles^8^, robotics^9,10^, and robot-assisted person following^10^. In VOT, a fundamental challenge is to learn an appearance model from the initial state of a target object, which is essential for locating the target object in subsequent frames^6,7^. This challenge becomes particularly pronounced with the presence of similar appearance distractors^6^.

A reliable visual object tracker is vital for the effectiveness of a vision-based drone-person following system in the face of numerous challenges. To train a robust tracker capable of excelling in diverse scenarios, it must be exposed to various challenging tracking scenarios. In recent years, several large-scale object tracking datasets have been released, such as UAV123^11^, OTB100^12^, VOT2018^13^, TrackingNet^14^, LaSOT^15^, GOT-10k^16^, and LaTOT^17^, which cover diverse real-world tracking challenges. However, none of these datasets specifically cover person tracking in a uniform appearance environments. Such settings are common in regions like the Gulf and many parts of Asia and pose unique tracking challenges due to similar clothing. Introducing a dataset for uniform appearance tracking can be a valuable addition to the computer vision community. A recent study^18^ has introduced a dataset named PTUA, which focuses on ground robot-person tracking in a uniform dressing environment scenario. However, this dataset has several limitations in the settings chosen while recording it. Firstly, it only considers a maximum person density of four, which does not reflect a truly crowded scenario. Secondly, the dataset was captured in a controlled environment, which does not accurately simulate the challenges of real-world tracking scenarios. Thirdly, the dataset does not simulate scenarios where a person may be imitating intruder behavior, such as moving quickly to evade a robot which are essential factors in assessing the robustness of a tracking algorithm in a person tracking scenario.

**Fig. 1:**
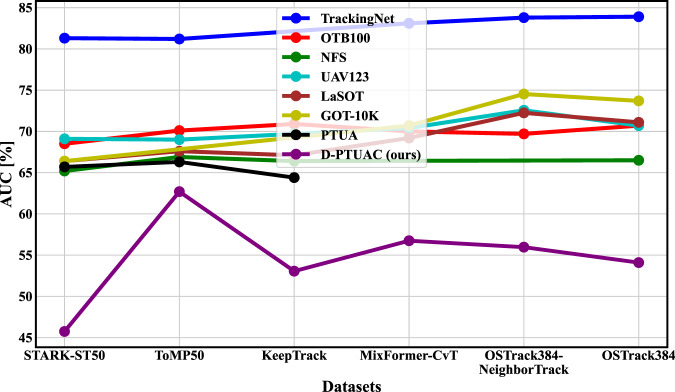
A visual performance evaluation encompasses a comparative analysis of six state-of-the-art trackers, specifically STARK-ST50^25^, ToMP50^22^, KeepTrack^46^, MixFormer-CvT^37^, OSTrack384-NeighborTrack^47^, and OSTrack384^41^. This evaluation is carried out across seven distinct datasets, followed by a direct comparison with our proposed D-PTUAC dataset. It is noteworthy that the results underscore a noticeable decline in the performance of the state-of-the-art trackers when applied to our D-PTUAC dataset in sharp contrast to their performance on the other seven datasets.

In scenarios that involve tracking individuals using drones in uniform appearance crowd, the task of maintaining the trajectory of a designated target becomes notably challenging. This challenge arise from the complexities introduced by drone-based capturing and the substantial presence of uniform appearance distractors. The combined impact of these challenges creates obstacles in effectively tracking the intended object, thereby rendering it more intricate compared to tracking tasks in other datasets, as visually illustrated in Fig. 1, and as comprehensively discussed in *“Technical Validation”* section.

**Fig. 2:**
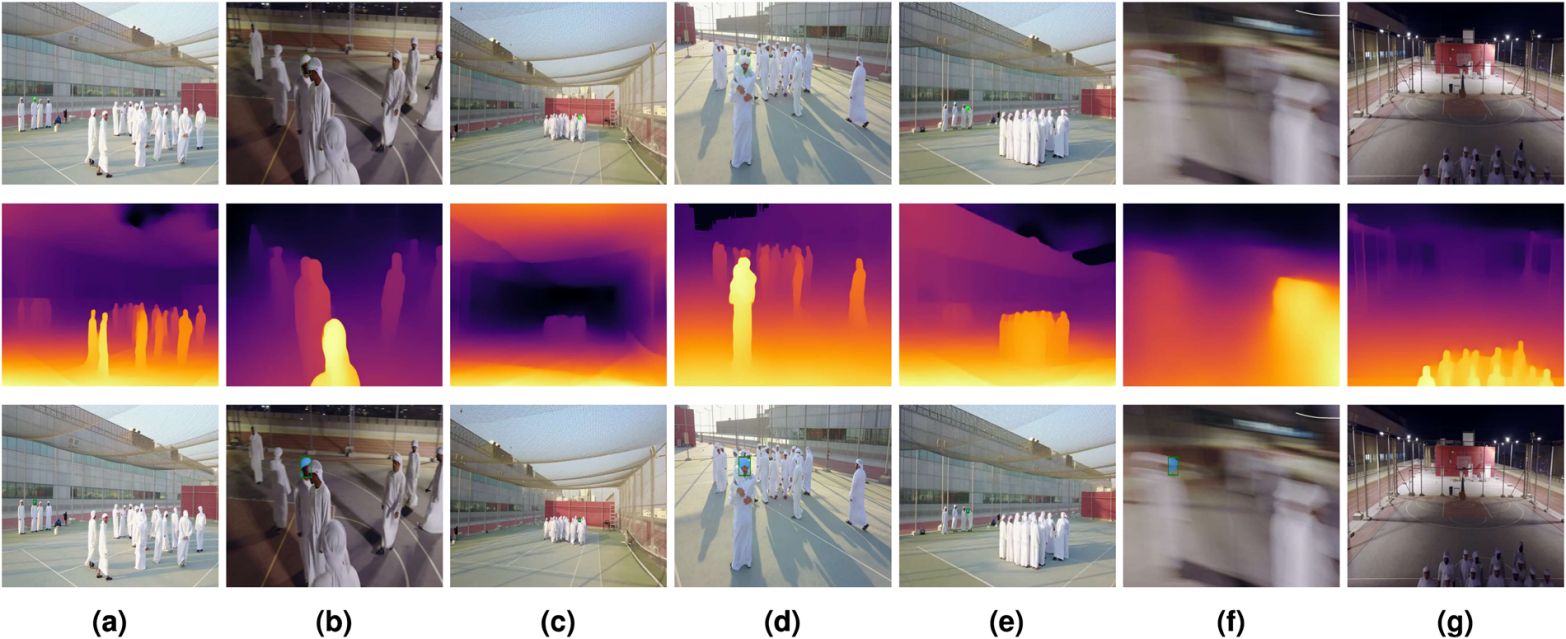
Selected samples from the proposed dataset highlighting challenging attributes (IV, BC, UF, OCC, PV, MB, FM, OV, AAC, ARC, DEF, ROT, CD, SV, ST, LT) through a structured layout: Row 1 showcases RGB sample images, Row 2 presents depth sample images, and Row 3 displays Segmentation masks sample images. The columns within the figure showcase samples that encompass multiple attributes, where Column (**a**) features LR, IV, BC, and UF, Column (**b**) includes OCC, BC, IV, and UF, Column (**c**) portrays IV, BC, and UF, Column (**d**) demonstrates OCC, BC, IV, UF, and PV, Column (**e**) encompasses DEF, LR, BC, IV, and UF, Column (**f**) showcases MB and FM, and Column (**g**) encompasses OV. These images emphasize the importance of developing robust drone-person tracking methods.

**Fig. 3:**
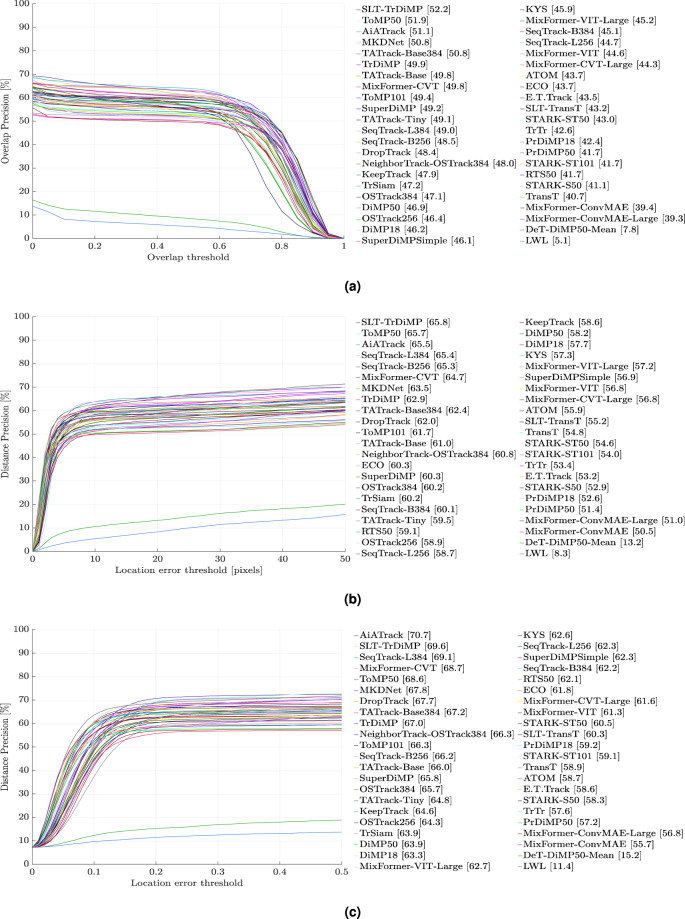
Evaluation results of 44 state-of-the-art pretrained trackers on D-PTUAC on videos with LR and BC attributes using, (**a**) Success Rate, (**b**) Precision Rate, and (**c**) Normalized Precision Rate. Please zoom for better clarity.

To address the above gaps, we introduce the Drone-Person Tracking in Uniform Appearance Crowd (D-PTUAC) dataset^19^ for uniform-clothed crowds. The dataset also stands out in having a target person behaving as an intruder to single them out from the crowd. The sequences were collected by controlling a camera-equipped drone, specifically DJI Mavic 3 Pro (https://www.dji.com/ae/mavic-3-pro) with a wireless manual controller using DJI GO 4 Android application (https://www.dji.com/ae/downloads/djiapp/dji-go-4) to follow a target person among the crowd wearing the same attire. To enhance the diversity of the dataset, we performed the collection in different challenging scenarios such as Uniformity (UF), Abrupt Appearance Change (AAC), Background Clutters (BC), Aspect Ratio Change (ARC), Scale Variation (SV), LR, Rotation (ROT), Pose Variation (PV), Occlusion (OCC), Out-of-View (OV), Short-Term (ST), Long-Term (LT), Motion Blur (MB), Fast Motion (FM), Illumination Variations (IV), Weather Conditions (WC), Crowd Densities (CD), Deformation (DEF), and Surveillance Settings (SS). Figure 2 illustrates sample images taken from the proposed D-PTUAC dataset. The evaluation of RGB trackers revealed a substantial performance decline, particularly in the presence of LR and BC, as illustrated in Fig. 3. This decline can be attributed to the nature of the dataset captured by a drone, where the subjects tend to be LR and wear clothing with a uniform appearance, intensifying the challenges of UF and BC. Additionally, to highlight the challenges in drone crowd uniform appearance tracking, we show that previous frameworks that rely on estimated depth fusion and segmentation fail on our dataset. For this purpose, we employed two frameworks: MiDaS for monocular depth estimation^20^ to generate RGB-D data and ViT-B SAM^21^ to generate segmentation masks for tracking. The dataset^19^ is available for access on Figshare at (10.6084/m9.figshare.24590568.v2).

## Methods

### Human subjects

Our study involving human subjects was approved by the Research Ethics Committee of Khalifa University of Science and Technology (IRB protocol number H23-029), ensuring adherence to ethical standards in research. Following this approval, we specifically targeted the Khalifa University community for participant recruitment, encompassing students, staff, faculty, and local residents in Abu Dhabi, United Arab Emirates. This effort successfully engaged approximately 40–50 subjects; all of whom voluntarily participated in the construction of the dataset. The Ethics office, in collaboration with the investigators, played a key role in disseminating detailed information about the study and obtaining informed written consent from all participants. The inclusion criteria for our participant cohort were clearly defined: individuals aged 18 years and above, of any sex, either UAE nationals or residents, who were capable of understanding and providing consent. Exclusion criteria included individuals below 18 years of age and those unwilling or unable to give consent. Importantly, participants were explicitly informed that their likenesses captured in videos and images would be shared as part of an open-access dataset, thus ensuring their full awareness and understanding of the extent of their involvement and how their data would be utilized in the research community.

### Proposed D-PTUAC dataset

To construct a benchmark dataset tailored for drone-person tracking scenarios, we conducted RGB video recordings using a DJI Mavic 3 Pro drone. During these recordings, the drone was manually operated to track and follow a designated individual within a group. This approach allowed us to capture a range of typical drone navigation characteristics and challenges, including ego-motion, MB, and occurrences of OCC. Subsequent sections will provide detailed insights into the dataset construction process.

#### Surveillance settings

The D-PTUAC dataset comprises videos for dynamic and static SS to simulate real-world scenarios. Sample frames extracted from these videos are visually depicted in Fig. 2. Below, we provide a comprehensive overview of the specific applications and dataset particulars pertaining to these two distinct SS.

- Dynamic surveillance involves actively monitoring a particular subject or group of subjects using a moving drone. The D-PTUAC dataset features 88 videos specifically recorded for dynamic surveillance. Participants were instructed to walk from point A to point B while the drone captured their movement from both the front and back views, although not simultaneously. The drone closely monitors the subject by flying a few meters ahead of them, and user cooperation is not necessary, as the drone is designed to follow the subject’s movements via manual control using the DJI GO application.
- Static surveillance involves using a static drone to monitor an area or event without a specific focus on any particular subject or object. The D-PTUAC dataset includes 50 videos captured for static surveillance, each featuring participants instructed to move around, engage in discussions, or walk within a designated area while the drone captured the entire scene. Unlike dynamic surveillance, the drone’s movement is kept static, and its objective is to record the events of the specified region rather than monitor specific individuals. This approach simulates real-world surveillance scenarios, making the D-PTUAC dataset a challenging and valuable resource for drone-based tracking and understanding of human activities.

#### Dataset setup

The D-PTUAC dataset consists of twenty-four distinct settings, offering variation in terms of SS, angle of view, CD, and times of capture. The dataset was meticulously collected on an outdoor tennis court located at Khalifa University in Abu Dhabi, United Arab Emirates. The tennis court has dimensions of 20×10×4 meters.

The data collection spanned two seasons, Fall and Spring, with diverse crowds to enhance dataset diversity. In the Fall season, Crowd 1 videos were recorded during both morning and evening sessions, whereas in the Spring season, Crowd 2 videos were exclusively captured during the morning hours, except for one instance characterized by rainy weather conditions. The dataset introduces three CD categories, namely *sparse*, *medium*, and *compact*. For each CD category, participants were recorded from both front and back views, resulting in a total of 87 videos recorded in the morning for each CD and SS, along with an additional 51 videos captured in the evening. It is worth noting that the morning videos possess high illumination, while the evening videos exhibit relatively lower illumination levels. To ensure sufficient lighting in the evening videos, floodlights were employed.

**Fig. 4:**
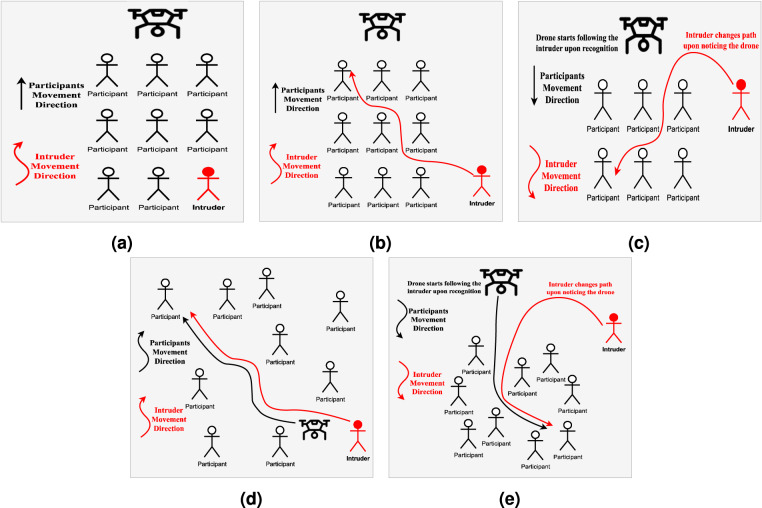
Visual illustration of the scenarios employed for D-PTUAC dataset collection. (**a**) Scenario 1 (S1), (**b**) Scenario 2 (S2), (**c**) Scenario 3 (S3), (**d**) Scenario 4 (S4), and (**e**) Scenario 3 (S5).

Figure 4 visually illustrates scenarios involving a crowd with an intruder among them. These scenarios are constructed under the assumption that the individual to be tracked is an intruder, and the objective is for the drone video tracker to effectively follow their movements as they navigate through and within the crowd, occasionally attempting to evade the tracker’s surveillance.

1. Scenario 1 (S1): The uniform appearance crowd moves in a straight line from point A to B, while the intruder, present within the crowd, attempts to confuse the tracker by moving in an overlapping and zigzag path.
2. Scenario 2 (S2): The uniform appearance crowd moves in a straight line from point A to B, and the intruder joins the crowd at a later stage. The intruder then moves in an overlapping and zigzag path to confuse the tracker.
3. Scenario 3 (S3): The uniform appearance crowd moves in a straight line from point A to B, and the intruder initially moves in a circular path towards the drone. Upon noticing the drone, the intruder quickly joins the crowd and continues to move in an overlapping and zigzag path to confuse the tracker.
4. Scenario 4 (S4): The uniform appearance crowd moves randomly within the tennis court area, while the drone 5tries to follow the intruder. The intruder attempts to overlap with the crowd to confuse the tracker and hide. This scenario represents a dynamic SS, as the drone follows the intruder throughout the video.
5. Scenario 5 (S5): The uniform appearance crowd is instructed to move randomly within a confined area of the tennis court. Meanwhile, the intruder moves in a circular path towards the drone. Once the intruder notices the drone, they immediately join the crowd and attempt to overlap with them to confuse the drone tracker and evade detection. The drone tries to follow the intruder throughout the video, while the intruder tries to hide within the crowd.

The D-PTUAC dataset contains repeated videos, and this is intentional due to the division of each crowd into groups of intruders. Each group participated in one of the five scenarios mentioned earlier, leading to multiple videos of the same scenario but with different intruders. The collection process of the D-PTUAC dataset is described in Algorithm 1.

##### Algorithm 1

Algorithmic Overview of D-PTUAC Dataset Collection Process.

The D-PTUAC dataset consists of a range of visual factors, including PV, IV, OCC, and LR, as depicted in Fig. 2. Each video in the dataset contains approximately 40–50 subjects for the two crowds, with 15–30 subjects per combination of CD, angle of view, drone SS (static or dynamic), and time of capture.

In terms of subject demographics, all individuals captured in the videos fall within the age range of 20 to 35 years. Additionally, a subset of videos includes two participants who exceed the age of 35 years. For each specific combination of settings, a single group of 2–5 intruders appears in only one video, resulting in a total of 138 videos across the twenty-four combinations. Detailed statistics of the D-PTUAC dataset can be found in Table 1, which includes over 76 K frames for dynamic SS and over 44 K frames for static SS.

**Table 1:** Comprehensive Statistics of the D-PTUAC Dataset.

Characteristic	Surveillance Setting		
	Dynamic	Static	Total
Videos	88	50	138
Subjects	15–30	15–30	15–30
Mean (secs.)	28.98	29.97	29.48
Min. (secs.)	13.3	8.67	8.67
Total Duration (mins.)	42.5	24.98	67.47
Total Frames	76,499	44,955	121,454

The dataset also encompasses high-resolution gallery images of each subject captured in constrained settings using a 12-megapixel smartphone. The video scenarios were captured at a frame rate of 30 Frames Per Second (FPS) and a resolution of 3840×2160 pixels using a DJI Mavic 3 Pro drone. These gallery images were taken in optimal lighting conditions and encompass four distinct poses. These images serve multiple purposes, including the development of a facial identification system capable of recognizing intruders in aerial images, even when the facial area is limited to only a few pixels. Furthermore, these images are integral to a comprehensive tracking framework. Initially, a face detector identifies the first bounding box of a specific person, which is then confirmed by a face recognizer. This information is subsequently passed on to the tracker to initiate the tracking process.

#### Videos annotations

In the annotation process for the D-PTUAC dataset, the heads of the subjects were selected as the most suitable body part for annotation. This choice was made to address the challenges presented by subject overlap and OCC. Precisely defined bounding boxes were employed to encompass the visible region of the heads, taking into consideration the perspective from the drone.

To assess the quality of head annotations in the D-PTUAC dataset, a comparison was made against full-body annotations using twenty video sequences and a pretrained ToMP50 tracker^22^. The head-tracking success rate achieved 55.29%, significantly outperforming the full-body tracking success rate of 11.09%. This outcome underscores the superiority of head annotations for the specific tracking tasks in this dataset.

The annotation process for the dataset was meticulous, involving a team of experienced individuals with expertise in VOT. Manual annotation was performed using the Computer Vision Annotation Tool (CVAT) (https://www.cvat.ai/) with precise attribute labels assigned. The process underwent three stages of scrutiny and refinement to ensure the annotations’ high quality. Several challenges were encountered during the annotation process, including small head sizes in the video frames, OCC, MB, LR, UF, and BC. Addressing these challenges necessitated re-annotation for approximately 80% of the dataset.

**Fig. 5:**
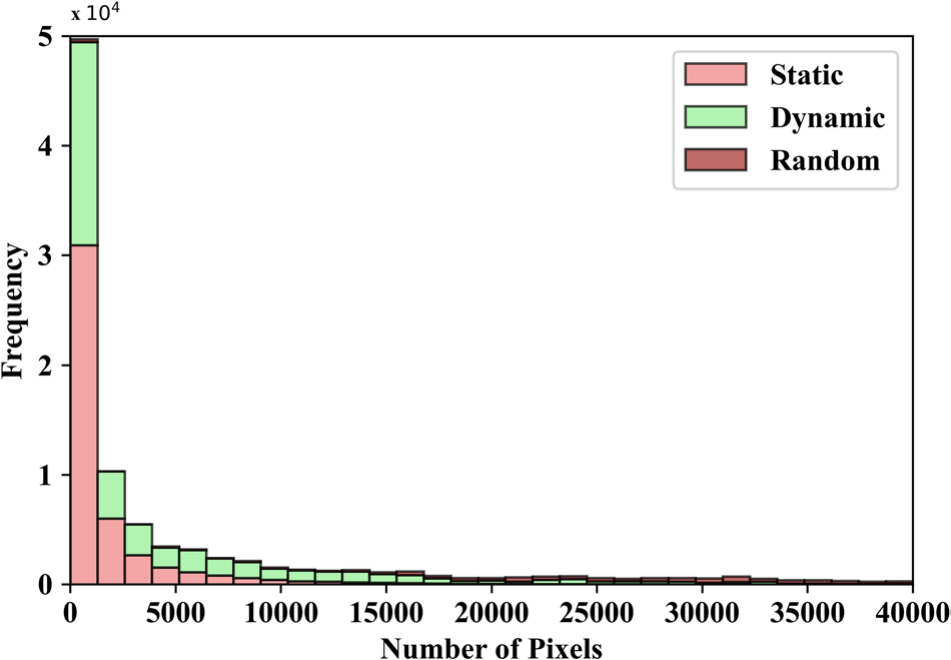
The histogram represents the distribution of ground truth annotated heads based on their size. It can be observed that a significant portion of the ground truth images have less than 4,000 pixels, which is equivalent to images smaller than 64×64 pixels.

Annotating only the head of the target person in the D-PTUAC dataset results in small bounding boxes of size 64×64 pixels, as depicted in Fig. 5, leading to challenging LR samples. These small targets lack sufficient appearance information and pose difficulties for deep networks, which produce weak features when directly processing LR regions. Enlarging the regions introduces blur and sawtooth artifacts, compromising LR image representation and increasing computational costs. Additionally, when LR objects occupy a small portion of the image, they are vulnerable to interference from background objects and noise. These combined challenges impede the localization and discrimination capabilities of general visual tracking networks when dealing with LR objects^17^.

#### Dataset tracking attributes

**Fig. 6:**
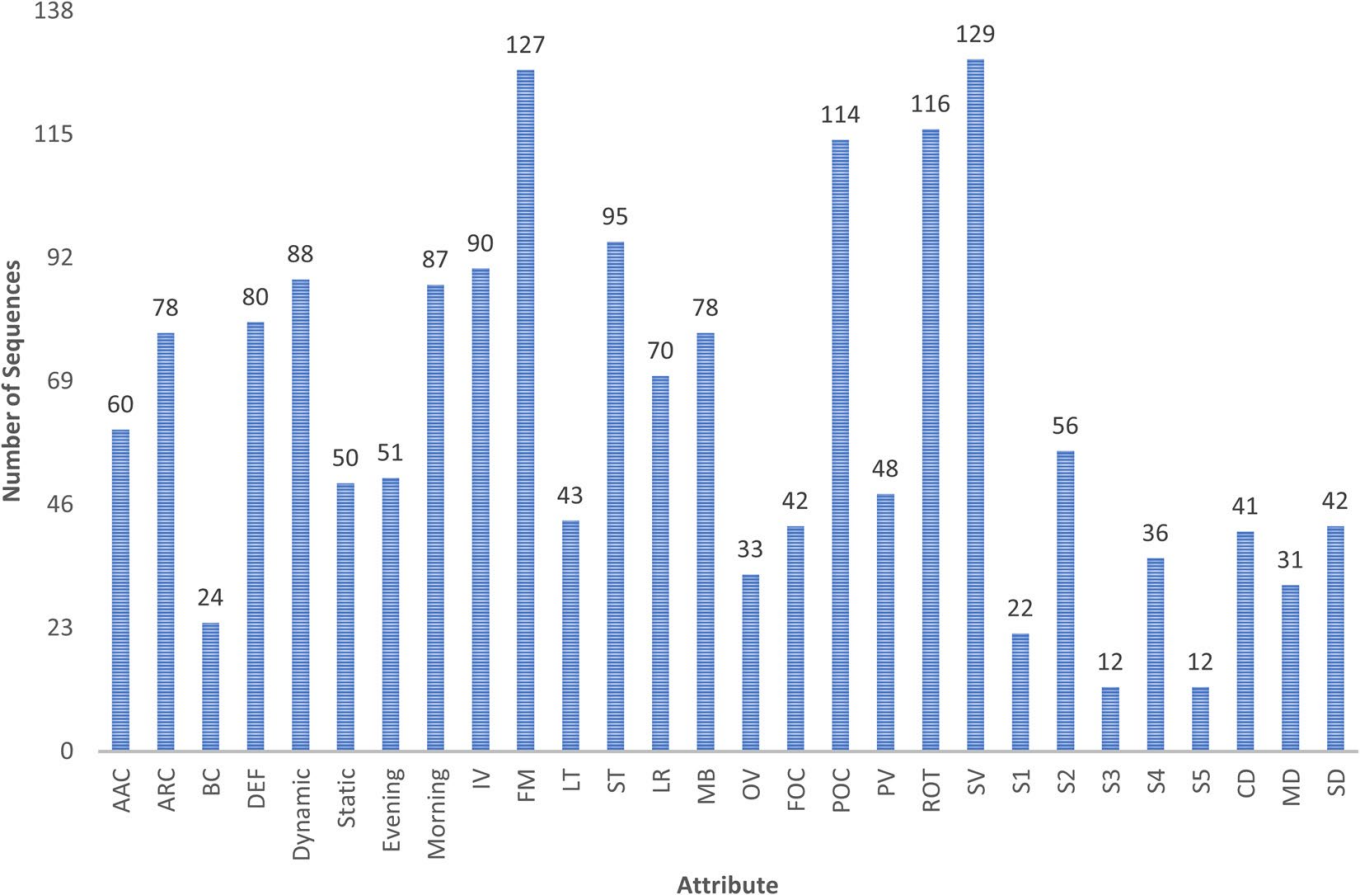
Distribution of Sequences Across Each Attribute within the D-PTUAC Dataset.

Drone-person tracking scenarios often present various challenging factors, many of which have been intentionally incorporated into the scenarios described in the *“Dataset Setup”* section. The tracking attributes in the D-PTUAC dataset, as shown in Fig. 6, can be classified into two groups: controlled and implicitly inherited. This study focuses on four crucial video-level controlled attributes relevant to aerial environments and significantly impact tracking algorithm performance in aerial captured video sequences. These attributes are summarized as follows:

1. Abrupt Appearance Change: It describes sudden and significant changes in the appearance of the tracked object. In the scenarios discussed earlier, such those in S3 (Fig. 4a) and S5 (Fig. 4e), where the intruder executes zigzag movement and blending into the crowd can cause AAC. This results in instances where the initially tracked region corresponds to the front head but subsequently shifts to the back head.
2. Crowd Density: It represents the proximity and density of individuals in a crowd. The dataset categorizes CD into three levels: *sparse* (more than 1-meter distance), shown in Fig. 2a, m*edium* (1-meter distance), shown in Fig. 2c, and *compact* (shoulder-to-shoulder proximity), shown in Fig. 2e.
3. Surveillance Settings: It refers to dynamic and static SS. Dynamic surveillance, with 88 videos, involves capturing subjects’ movements while the drone follows them, while static surveillance, with 50 videos, documents events in a designated area without actively tracking individuals.
4. Illumination Variation: It denotes significant changes in lighting conditions within a scene. In the drone-person following scenario, IV can arise from various sources such as the sun’s position, artificial lights, shadows, and reflections. The dataset captures videos in different lighting conditions, including morning, evening, and rainy weather. Examples are shown in Fig. 2a,b.

Additionally, 14 implicit attributes in the dataset were inherited from the nuisance and distraction factors. These attributes arise from factors that were not explicitly controlled or introduced by humans but were inherent in the dataset recordings. They are briefly described below:

1. Uniformity: The D-PTUAC dataset exhibits a unique characteristic wherein all individuals, including the target and distractors, wear a white dress and headscarf throughout the recording, as depicted in Fig. 2a–e. This feature distinguishes our proposed dataset from others, such as the recent robot-person tracking dataset^18^, which includes a similar attribute but with fewer people. In our dataset, scenes consist of 15–30 people, making individuals appear as moving blobs without visible leg movements. The scenes also feature two to five intruders who subsequently join the crowd, augmenting the dynamic nature and appearance of the scene.
2. Fast Motion: It occurs when the tracked object or the drone moves quickly, challenging the tracker to keep up.
3. Motion Blur: It arises from the drone or target’s movement, causing blurred frames, as illustrated in Fig. 2f.
4. Pose Variation: It captures the variability of human poses, including actions like running or hugging, resulting in significant pose changes between consecutive frames, as shown in Fig. 2a,d.
5. Scale Variation: It represents changes in the object’s size from the first frame to the current frame is outside the range of [0.5, 2], particularly in static scenarios where the target moves closer or farther from the drone as shown in Fig. 2a–d.
6. Background Clutter: It occurs when the target’s appearance resembles the background, leading to challenges in accurate differentiation, as shown in Fig. 2a–g.
7. Low Resolution: It describes the characteristics of the target object in the video frames, specifically referring to the tracked person’s head in our D-PTUAC dataset. To determine LR, we follow the method proposed in^17^, which calculates the object’s relative size by dividing its bounding box area by the image area in each frame. The average relative size is then computed across all frames within each video sequence. We set the average relative size threshold at 1%, as suggested in^17^. However, to prevent misclassification of larger object sequences as LR sequences, we incorporate the concept of average absolute size, with a threshold set at 22×22 pixels. For a video sequence to be classified as LR, both the average absolute and relative size must be below these thresholds. This dual-criteria approach enhances the accuracy of LR sequence identification in our study.
8. Rotation: It happens when the target deliberately conceals themselves within the crowd after being detected by the drone.
9. Aspect Ratio Change: The bounding box aspect ratio falls outside the specified range of [0.5, 2].
10. Deformation: The target undergoes deformations and changes in shape during the tracking process, as depicted in Fig. 2e.
11. Occlusion - Partial Occlusion (POC)/Full Occlusion (FOC): It arises when parts or the entire target is obstructed by objects or people in the scene, as depicted in Fig. 2b–d.
12. Out of View: It refers to a situation where the target fully leaves the camera field of view, as shown in Fig. 2g.
13. Short-Term Videos: It refers to a sequence length of less than 1,000 frames. Our dataset contains 95 ST videos.
14. Long-Term Videos: It refers to a sequence length of more than 1,000 frames. Our dataset contains 43 LT videos.

### Frameworks for using RGB-D and segmentation based trackers

#### RGB-D framework

To comprehensively evaluate different categories of trackers, it is important to consider RGB-D trackers that rely on both RGB and depth data for fusion algorithms. However, since the D-PTUAC dataset is captured solely with an RGB camera, we have developed a framework to generate depth information from RGB data.

The RGB-D framework utilizes monocular depth estimation techniques, specifically leveraging the MiDaS network^20^. Specifically, we have employed the DPT-Swin2-Tiny-256 network, which balances accurate depth estimation and real-time inference on embedded devices, achieving a framerate of 90 FPS^20^. This choice is particularly important for deploying the framework on resource-constrained systems such as drones.

#### Segmentation mask generation framework

Given the uniqueness of our dataset, a segmentation model capable of generating masks for various objects, with a focus on the head in our case, is required. To address this, we have utilized the SAM model^21^, which has been trained on a large dataset of over 1 billion masks derived from 11 million licensed and privacy-respecting images. The extensive training enables the SAM model to generalize well and accurately segment specific targets, such as the head of the person of interest. Among the available options in the SAM model, we have chosen the ViT-base model due to its lightweight nature in terms of parameters and floating point operations (FLOPs), as well as its fast inference speed compared to other models discussed in the paper^21^.

## Data Records

The D-PTUAC dataset^19^ has been made available for public download through Figshare at (10.6084/m9.figshare.24590568.v2). Access to the data does not necessitate any registration process. The dataset occupies a combined storage of 15.01 gigabytes. An elaboration of the folder arrangement encompassing the dataset and its pertinent files is provided below.

### Folder structure

**Fig. 7:**
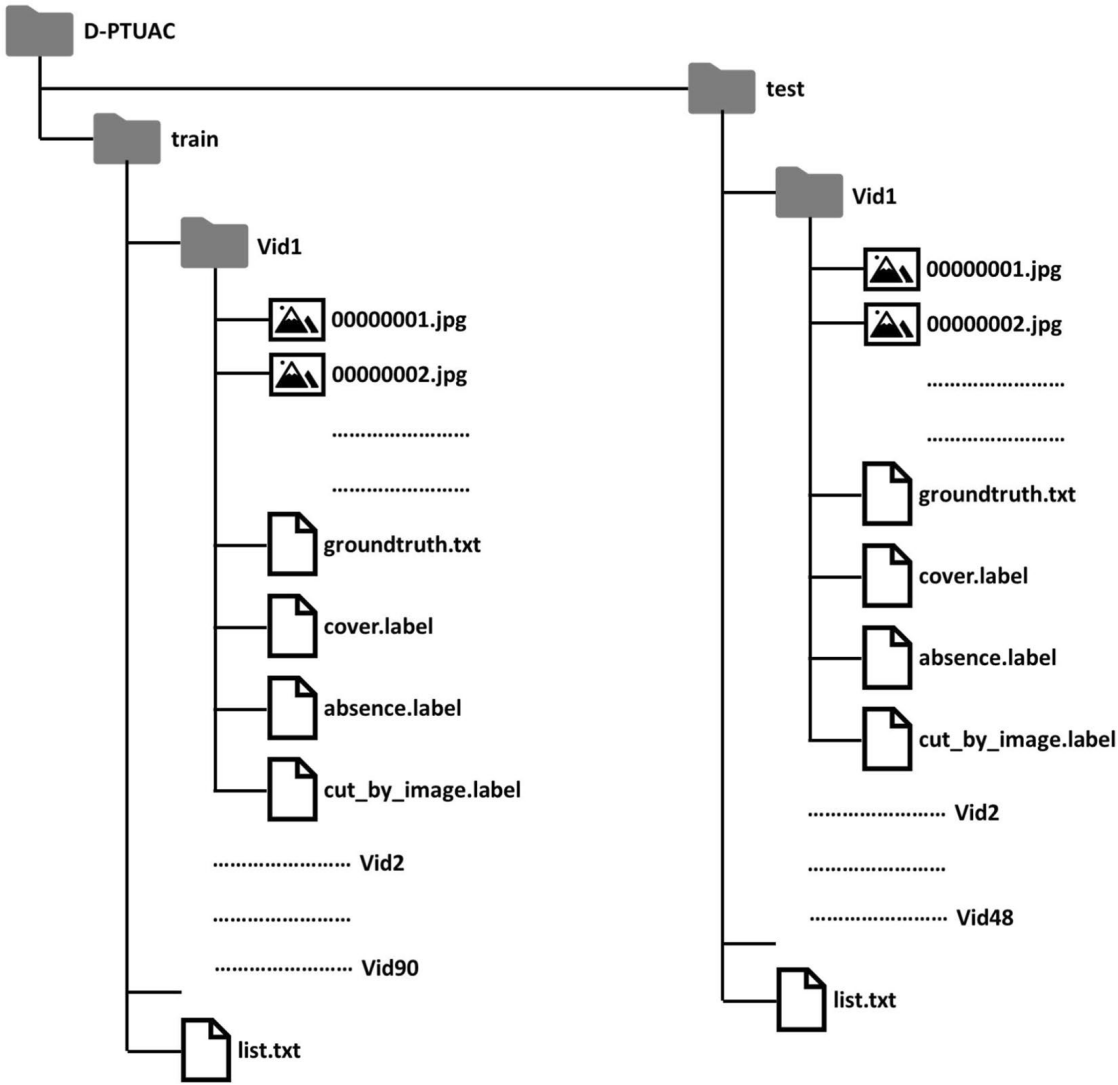
The folder structure of the D-PTUAC dataset.

An overview of the D-PTUAC dataset is given in Fig. 7. The dataset’s root directory, labeled *“D-PTUAC”*, comprises two subsidiary directories: *“train”* and *“test”*. Within the *“train”* subdirectory, there is a file named *“list.txt”*, listing video directories and 90 directories with video frames and four “.txt” files defined as follows: *“groundtruth.txt”* forms an *N*×4 matrix, indicating object locations as *[xmin, ymin, width, height]*. *“cover.label”* is an *N*×1 array, representing object visible ratios categorized from 0 to 8. *“absence.label”* is a binary *N*×1 array indicating object presence, and *“cut_by_image.label”* indicates if an object is cut by frame boundaries in each video frame. The same structure is followed in the “test” subdirectory, where a file named *“list.txt”* lists video directories along with 48 directories containing video frames, along with the four *“.txt”* files.

In the dataset, each video frame contained within the *“Vid”* directories is designated with an eight-digit numerical sequence that incrementally advances as the video unfolds. This sequence is followed by the *“.jpg”* extension, indicating the image data type. Pertinent annotations and essential information concerning the object of interest within each video are located within the corresponding video directory. For the structuring of annotations, the D-PTUAC dataset adheres to the annotations style established by the GOT-10K^16^ dataset.

## Technical Validation

We conducted a comprehensive performance evaluation of existing state-of-the-art (SOTA) trackers on our proposed D-PTUAC dataset, which included attribute-wise analysis to test the trackers’ robustness against specific challenges. To further enhance the tracking performance, we finetuned 10 high-quality SOTA trackers on a training split of the D-PTUAC dataset. All experiments were conducted on a workstation equipped with one Nvidia GeForce RTX 3080 GPU, 11th Gen Intel 2.3 GHz CPU, 32GB RAM, and 8GB VRAM. We used the official source codes provided by the respective authors to implement all trackers.

### Evaluation metrics

To evaluate the trackers, we used the popular One-Pass Evaluation (OPE) protocols proposed by OTB^12^ and LaSOT^15^ to measure Success Rate (SR), Precision Rate (PR), and Normalized Precision Rate (NPR).

The *SR*, is calculated by taking the Intersection over Union (IoU) of the pixels between the tracker’s predicted bounding box, *boxP*, and the actual ground truth’s bounding box, *boxG*.

1 $$SR=\frac{| bo{x}^{G}\cap bo{x}^{P}| }{| bo{x}^{G}\cup bo{x}^{P}| }$$

Tracking algorithms are ranked based on their *SR*, which is determined by the Area Under the Curve (AUC) ranging from 0 to 1. A higher AUC indicates a better success rate for the tracker. The ranking is done from the worst to the best-performing tracker.

In general, the *PR* is computed as the distance between the center of the ground truth bounding box and the predicted bounding box generated by the tracker. The *PR* is defined as:

2 $$PR=| | bo{x}^{G}-bo{x}^{P}| {| }^{2}$$

The ranking of trackers is determined by varying threshold values from 0 to 20 pixels in this measure, and those with a higher *PR* are considered to have better performance.

To address the sensitivity of the *PR* measurement to image resolution and bounding box sizes, we incorporated the *NPR*, measurement. The calculation of *NPR* is as:

3 $$\begin{array}{l}W=diag\left(bo{x}_{x}^{G},bo{x}_{y}^{G}\right)\\ NPR={\left\Vert W\left(bo{x}^{G}-bo{x}^{P}\right)\right\Vert }^{2}\end{array}$$

This metric, denoted by *NPR*, normalizes the *PR* using the ground truth annotations, as described in^14^. Trackers are ranked based on the AUC for *NPR* values ranging from 0 to 0.5. A higher *NPR* score indicates better performance of the tracker.

### Baseline trackers

The evaluation of the dataset involved a careful selection of representative SOTA baseline trackers in highlighting the challenges posed by the proposed dataset. A total of 44 prominent trackers were included to ensure a comprehensive evaluation. These trackers were chosen from different categories, including Discriminative Correlation Filters (DCF)-based trackers like ATOM^23^ and DiMP^24^, hybrid transformer-based trackers like STARK^25^, and TATrack^26^ which combine Siamese networks and transformer networks for improved feature discrimination, and the DCF-based RGB-D tracker DeT^27^ that extends the DiMP tracker^24^ to incorporate depth information. Segmentation-based trackers such as RTS^28^ were also included in the evaluation.

### Evaluation protocols

The evaluation protocol is designed to assess the following aspects: [label = ()]

- Overall Performance on the Testing Set: Comparing the performance of 44 SOTA trackers on the D-PTUAC testing set before and after finetuning.
- Drone Surveillance Settings (Multi-scale) Performance: Assessing trackers in dynamic and static drone scenarios to understand their capabilities and limitations under different operational conditions, including object tracking during drone movement and challenges such as MB, FM, OCC, and changing trajectories. Evaluating trackers in static drone settings provides insights into their performance in the presence of multiple uniform appearance distractors.
- Scenario Performance: Conducting individual evaluations for each scenario depicted in Fig. 4 to analyze the tracker’s performance under different intruder behaviors, such as circular paths (Fig. 2c,e), or attempts to blend in with the crowd (Fig. 2a).
- Crowd Density Performance: Performing separate evaluations for sparse, medium, and compact CD levels to understand the impact of uniform appearance distractors on tracker performance, as they act as dynamic obstacles with similar appearances.
- Different Daytime Performance: Evaluating the performance of trackers in morning and evening scenarios to assess their adaptability and robustness under varying lighting conditions and environmental changes.
- Attribute Evaluation: Using trackers to assess distinct attributes exhibited in the videos, enabling in-depth analysis of tracker performance related to specific attributes.

For sections “Drone Surveillance Settings (Multi-scale) Performance”, “Scenario Performance”, “Crowd Density Performance”, “Different Daytime Performance”, and “Attribute Evaluation”, we specifically selected 10 SOTA trackers that underwent finetuning based on the results presented in Tables 3, 4. A comparison is then made between these chosen trackers for each evaluation protocol. The objective behind these evaluations is to conduct a comprehensive analysis of the trackers’ performance while also gaining insights into the impact of various scenarios and attributes on their effectiveness.

### Training/Testing split

The D-PTUAC dataset was split into training and testing sets. The training set consists of 90 videos, while the testing set consists of 48 videos. The training set contains approximately 78 K frames, while the testing set contains around 42 K frames. A comprehensive comparison of the training and testing sets of D-PTUAC is presented in Table 2. The analysis shows that the minimum frames, mean frames, median frames, and maximum frames exhibit similarity between these two subsets. Additionally, Fig. 9 demonstrates that the ratios of sequences across all attributes and settings are also similar. These findings, derived from both Table 2 and Fig. 9, provide evidence of the consistency and coherence of our training/testing split.

**Table 2:** Comparison between training and testing sets of D-PTUAC.

	Video	Min frames	Mean frames	Median frames	Max frames	Total frames	Total duration (min.)
D-PTUAC-training	90	343	876	816	1787	78,878	43.82
D-PTUAC-testing	48	260	887	835	1679	42,576	23.65
D-PTUAC	138	260	880	818	1787	121,454	67.47

#### Overall Performance on the testing set

**Fig. 8:**
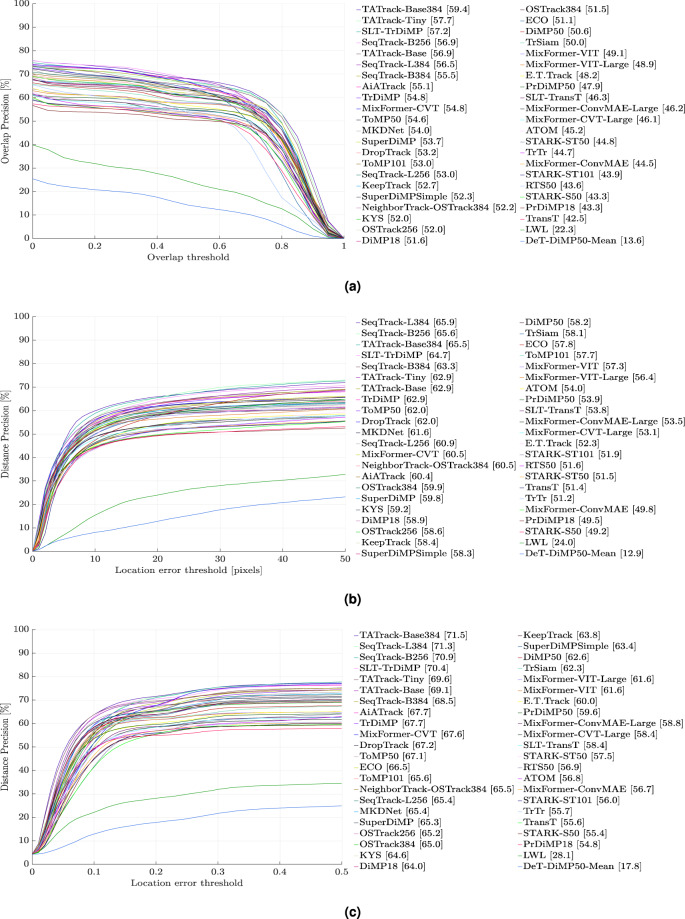
Evaluation results of 44 pretrained models on D-PTUAC testing set using, (**a**) SR (%), (**b**) PR (%), and (**c**) NPR (%). Please zoom for better clarity.

To conduct a comprehensive analysis, we evaluate the performance of 44 pretrained trackers on the testing set of D-PTUAC, as depicted in Fig. 8. Among these trackers, 24 are listed in Table 3, which showcases their performance in the pretrained state. Additionally, we present 20 trackers in Table 4, which demonstrates their performance before and after finetuning on the training set of D-PTUAC, allowing us to assess the impact of our training set on tracker performance. It is important to note that no changes were made to the hyperparameters of these 20 trackers.

**Fig. 9:**
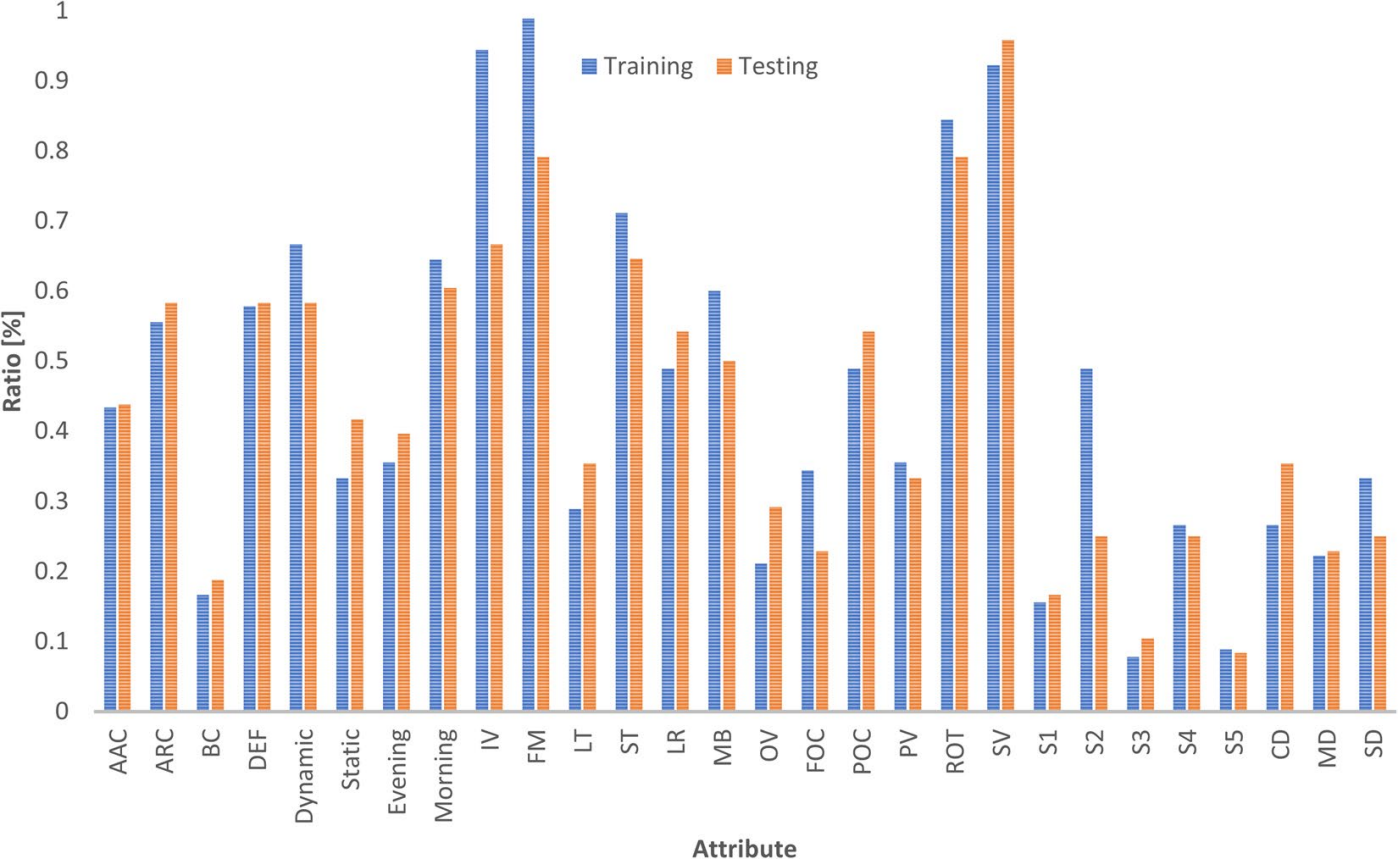
Comparison of sequence distribution in each attribute between training and testing sets.

**Table 3:** Comparative results of pretrained trackers on D-PTUAC testing set.

Tracker	SR (%)	PR (%)	NPR (%)
TATrack-Base (2023)^26^	56.88**	62.94***	69.12***
TATrack-Tiny (2023)^26^	57.65*	62.94***	69.60**
SeqTrack-L384 (2023)^29^	56.47***	65.94*	71.33*
SeqTrack-B384 (2023)^29^	55.45	63.31**	68.46
SeqTrack-L256 (2023)^29^	53.01	60.90	65.37
SLT-TranT (2022)^36^	46.29	53.80	58.36
ToMP101 (2022)^22^	53.02	57.70	65.62
SuperDiMPSimple^32^	52.27	58.29	63.36
MixFormer-CvT-L (2022)^37^	46.14	53.13	58.42
MixFormer-ConvMAE (2022)^37^	44.53	49.77	56.72
MixFormer-ConvMAE-L (2022)^37^	46.16	53.51	58.76
MixFormer-ViT (2022)^37^	49.12	57.34	61.63
MixFormer-ViT-L (2022)^37^	48.94	56.35	61.63
KYS (2020)^40^	52.04	59.19	64.63
OSTrack256 (2022)^41^	52.04	58.63	65.23
ECO (2017)^42^	51.14	57.80	66.50
E.T.Track (2023)^43^	48.23	52.32	60.01
ATOM (2019)^23^	45.24	53.98	56.76
TrTr (2021)^30^	44.74	51.20	55.69
TransT (2021)^31^	42.55	51.37	55.56
STARK-ST101 (2021)^25^	43.86	51.89	56.00
STARK-S50 (2021)^25^	43.34	49.21	55.40
RTS (2022)^28^	43.62	51.63	56.91
LWL (2020)^44^	22.25	24.02	28.12

Trackers are ranked per metric, with the best indicated by *, second best by **, and third best by ***.

**Table 4:** Comparative results of pretrained versus finetuned trackers on D-PTUAC testing set.

Tracker	Mode	SR (%)	PR (%)	NPR (%)
TATrack-Base384 (2023)^26^	Pretrained	59.39*	65.49**	71.54*
	Finetuned	64.74*	69.77	79.59*
SeqTrack-B256 (2023)^29^	Pretrained	56.95***	65.60*	70.93**
	Finetuned	61.50	68.31	73.51
SLT-TrDiMP (2022)^36^	Pretrained	57.24**	64.70***	70.39***
	Finetuned	58.45	67.69	72.25
AiATrack (2022)^34^	Pretrained	55.13	60.37	67.71
	Finetuned	63.87	73.72*	76.41***
ToMP50 (2022)^22^	Pretrained	54.63	62.03	67.06
	Finetuned	62.70	69.95	74.70
TrDiMP (2021)^35^	Pretrained	54.84	62.85	67.66
	Finetuned	64.21**	73.06**	77.54**
TrSiam (2021)^35^	Pretrained	49.98	58.12	62.30
	Finetuned	61.55	69.16	74.72
MKDNet (2023)^17^	Pretrained	54.03	61.60	65.36
	Finetuned	58.73	65.54	71.02
SuperDiMP^32^	Pretrained	53.67	59.84	65.26
	Finetuned	64.15***	70.85***	76.41***
DiMP50 (2019)^24^	Pretrained	50.60	58.16	62.63
	Finetuned	55.38	63.70	67.97
DiMP18 (2019)^24^	Pretrained	51.62	58.95	63.96
	Finetuned	62.23	68.60	75.93
PrDiMP50 (2020)^33^	Pretrained	47.94	53.93	59.60
	Finetuned	56.78	61.43	69.15
PrDiMP18 (2020)^33^	Pretrained	43.26	49.45	54.83
	Finetuned	45.33	49.90	58.21
DropTrack (2023)^45^	Pretrained	53.24	61.98	67.18
	Finetuned	58.21	63.99	69.63
MixFormer-CvT (2022)^37^	Pretrained	54.77	60.51	67.61
	Finetuned	56.75	64.24	70.79
KeepTrack (2021)^46^	Pretrained	52.71	58.44	63.82
	Finetuned	53.06	58.59	64.34
OSTrack384-NeighborTrack (2022)^47^	Pretrained	52.22	60.48	65.51
	Finetuned	55.97	64.70	68.32
OSTrack384 (2022)^41^	Pretrained	51.47	59.88	65.00
	Finetuned	54.10	60.38	66.51
STARK-ST50 (2021)^25^	Pretrained	44.80	51.51	57.45
	Finetuned	45.75	52.33	58.29
DeT-DiMP50-Mean (2021)^27^	Pretrained	13.57	12.87	17.82
	Finetuned	19.53	17.94	23.03

Trackers are ranked per metric, with the best indicated by*, second best by**, and third best by***.

The analysis of the results reveals that algorithms that combine the Siamese network and Transformers, such as TATrack^26^, SeqTrack^29^, and ToMP^22^, demonstrate a higher level of robustness in performance. These algorithms effectively leverage the advantages of capturing contextual information and addressing LT dependencies, which are crucial for accurate tracking. However, it is worth noting that certain algorithms that combine the Siamese network and Transformers, including STARK^25^, TrTr^30^, and TransT^31^, fail to achieve satisfactory results on our dataset. A similar pattern is observed in DCF trackers, such as DiMP18^24^, SuperDiMP^32^, PrDiMP18^33^, and ATOM^23^. While SuperDiMP^32^ demonstrates robust performance by employing effective scale regression techniques and online learning strategies, ATOM^23^ falls short in achieving desirable results.

Specifically, the results indicate that TATrack-Base384^26^ achieved the highest performance with values of 59.39% for SR and 71.54% for NPR. Regarding PR, SeqTrack-B256^29^ outperformed other trackers, achieving a PR of 65.60%. Upon finetuning, a similar trend was observed where TATrack-Base384^26^ continued to demonstrate its effectiveness, yielding the best results with values of 64.74% for SR and 79.59% for NPR. Notably, AiATrack^34^ achieved the highest PR of 73.72%.

RGB-D and segmentation-based trackers, such as DeT^27^ and RTS^28^, face significant challenges and exhibit notable failures on our testing set. The homogeneous distribution of the crowd at the same distance from the camera leads to identical depth outputs for all individuals. Consequently, the depth data presents a similar appearance for the entire crowd, making it difficult for trackers to differentiate and accurately track the target person. This lack of depth variation hampers the tracker’s ability to distinguish between individuals, leading to the loss of track on the target and compromising performance in such scenarios. The presence of OCC and multi-scale targets further exacerbates the challenges faced by segmentation-based trackers. These trackers rely on pixel-level segmentation masks, which can be unreliable when multiple individuals in the crowd have similar appearances. This results in inaccurate tracking and compromised performance on our testing set.

Upon analyzing the data presented in Table 4, it is clear that each of the 20 trackers studied shows a consistent enhancement in performance following the finetuning process using our training set. This notable improvement not only validates the effectiveness of our training set but also emphasizes its critical importance in the context of drone-person following in scenarios involving a crowd with uniform appearance.

**Fig. 10:**
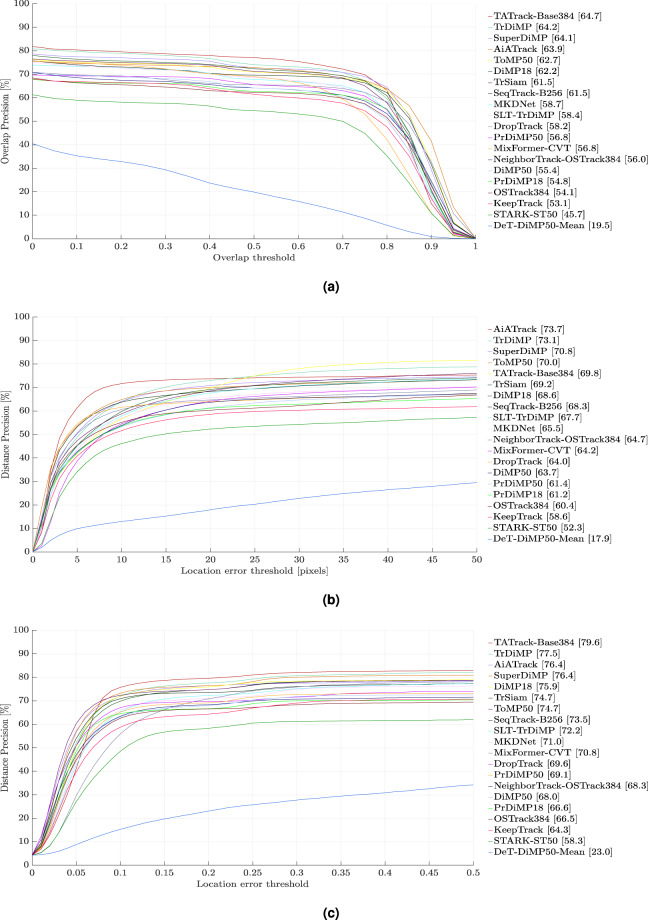
Evaluation results of 20 finetuned models on D-PTUAC testing set using, (**a**) SR (%), (**b**) PR (%), and (**c**) NPR (%). Please zoom for better clarity.

Additionally, Fig. 10 provides a visual representation of the comparatively limited performance exhibited by the 20 finetuned SOTA trackers when evaluated against the testing set. This diminished performance is indicative of the increased complexity and challenge inherent in the testing set, thereby underscoring the need for further advancements in tracker technology to effectively address such demanding scenarios.

#### Surveillance settings (Multi-scale) performance

As detailed in *“Surveillance Settings”* section, the D-PTUAC dataset comprises videos categorized into dynamic and static SS. In dynamic drone settings, the scale of the target’s bounding box remains relatively consistent due to the drone’s efforts to maintain a constant distance. However, in static drone settings, the target’s bounding box exhibits significant SV as the object moves closer to or farther away from the drone, resulting in multi-scale bounding boxes. This introduces challenges such as SV and necessitates tracking algorithms to effectively handle these changes and maintain accurate localization.

Based on the comparison provided in Table 5, AiATrack tracker^34^ demonstrates the highest performance in videos with multi-scale variations, particularly in static drone settings. Compared to the baseline tracker and the second best tracker, TrDiMP^35^, AiATrack achieves a performance improvement of 2.94%. Furthermore, TATrack-Base384^26^ is the top-performing tracker in dynamic drone settings, characterized by challenges such as MB and FM. In comparison to the baseline tracker and the second best tracker, SuperDiMP, TATrack-Base384 exhibits a performance enhancement of 1.08%.

**Table 5:** Comparative results of finetuned trackers on D-PTUAC testing set per SS.

	TATrack-Base384^26^	SeqTrack-B256^29^	SLT-TrDiMP^36^	MKDNet^17^	TrDiMP^35^	Super DiMP^32^	DropTrack^45^	ToMP50^22^	MixFormer-CvT^37^	AiATrack^34^
Static	54.70	56.10	54.39	54.94	58.15**	54.37	49.20	57.54***	52.61	59.86*
Dynamic	71.90*	65.36	61.35	61.44	68.54***	71.13**	64.65	66.39	59.71	64.64

Trackers are ranked per SR (%) metric, with the best indicated by *, second best by **, and third best by ***

#### Scenario performance

As outlined in Section *“Dataset Setup”*, the D-PTUAC dataset consists of five distinct scenarios that aim to simulate various intruder behaviors within a uniform appearance crowd. These scenarios are designed to replicate real-life situations where a law enforcement drone is deployed to track an intruder amidst a crowd with similar attire. Evaluating tracker performance in these scenarios is crucial for assessing their effectiveness in real-world applications. By evaluating the performance of the trackers on the D-PTUAC dataset, our objective is to gain insights into their capabilities and limitations when dealing with intruder tracking in uniform appearance crowd scenarios.

The benchmarking results presented in Table 6 reveal notable variations in tracker performance across different scenarios. Specifically, in scenarios S3 and S5, where the presence of appearance ambiguity challenges the trackers, a significant decline in performance is observed. The random behavior of the crowd in S5 further amplifies the performance degradation due to increased OCC. In both S2, S3, and S5, AiATrack^34^ outperforms other trackers, achieving performance improvements of 0.62%, 19.89%, and 35.18%, respectively, compared to the second-best performing trackers. Similarly, in scenario S1, characterized by high OCC levels as the intruder attempts to blend in with the crowd, SeqTrack-B256^29^ achieved highest performance of 80.45%. On the other hand, in scenario S4, where the drone closely follows the intruder, trackers exhibit a significant performance boost as the target remains within the drone’s field of view for most of the time. TATrack-Base384^26^ demonstrates the best performance in S4, surpassing the second best-performing tracker, SuperDiMP^26^, by 3.13%.

**Table 6:** Comparative results of finetuned trackers on D-PTUAC testing set per scenario.

	TATrack-Base384^26^	SeqTrack-B256^29^	SLT-TrDiMP^36^	MKDNet^17^	TrDiMP^35^	Super DiMP^32^	DropTrack^45^	ToMP50^22^	MixFormer-CvT^37^	AiATrack^34^
S1	77.1	80.45	71.33	69.55	75.80	79.40***	73.50	80.34**	67.33	73.19
S2	63.25	60.76	58.21	59.74	67.27**	64.01***	60.35	61.19	61.27	67.69*
S3	45.91**	41.19	41.09	38.04	42.11	42.29	40.10	43.87***	28.84	55.04*
S4	76.45*	66.93	67.67	68.28	72.70***	74.13**	64.18	68.87	63.38	58.73
S5	35.38	36.24***	27.86	29.48	28.69	31.71	22.20	39.65**	29.13	53.60*

Trackers are ranked per SR (%) metric, with the best indicated by *, second best by **, and third best by***.

#### Crowd density performance

The evaluation of trackers on different CDs, including sparse, medium, and compact, provides valuable insights into their capabilities and limitations in real-world surveillance scenarios. It allows for a comprehensive assessment of their performance in handling diverse crowd configurations, distinguishing targets from the background, and coping with OCC. Understanding the specific challenges and limitations associated with each density enables researchers and developers to enhance the trackers’ capabilities and address density-specific obstacles. Furthermore, benchmarking and comparing tracker performance across different densities facilitate informed decision-making for selecting suitable trackers based on specific CD requirements.

As shown in Table 7, there is a noticeable decline in performance among various trackers, such as SLT-TrDiMP^36^, SeqTrack-B256^29^, and AiATrack^34^, when transitioning from sparse to medium to compact CD. These variations align with an increase in OCC and the emergence of more complex BC. In contrast, in the sparse and medium CD levels, ToMP50^22^ achieves impressive AUC values of 72.15% and 68.94%, respectively. In compact CD scenario, SuperDiMP achieved slight performance improvement of 0.82% compared to the second-best performer, TrDiMP^35^.

**Table 7:** Comparative results of finetuned trackers on D-PTUAC testing set per CD.

	TATrack-Base384^26^	SeqTrack-B256^29^	SLT-TrDiMP^36^	MKDNet^17^	TrDiMP^35^	Super DiMP^32^	DropTrack^45^	ToMP50^22^	MixFormer-CvT^37^	AiATrack^34^
Sparse	65.46	68.45	68.35	69.48	70.35	70.80***	59.61	72.15*	64.54	71.67**
Medium	66.45	68.14**	62.44	57.25	67.08	61.59	58.73	68.94*	64.11	67.60***
Compact	68.96***	64.25	59.77	57.77	69.35**	69.92*	67.27	62.63	54.22	59.14

Trackers are ranked per SR (%) metric, with the best indicated by *, second best by **, and third best by ***

#### Daytime performance

The evaluation of trackers on morning and evening scenarios in SS offers multiple benefits. It provides valuable insights into their performance under varying lighting conditions, ensuring their adaptability to different times of the day. This evaluation also allows for the assessment of trackers’ robustness in handling challenges such as shadows, IV, and low light conditions specific to morning and evening environments. Additionally, it enables the analysis of potential temporal variations in tracking accuracy, aiding in the selection of trackers that exhibit consistent performance throughout the day.

As indicated in Table 8, all trackers performed better in the evening compared with the morning. A possible reason for a tracker performing worse in videos captured in the morning compared to the evening could be variations in lighting conditions. In the morning, the lighting may be softer, with lower contrast and potentially more shadows, making it challenging for the tracker to accurately detect and track objects. Additionally, the angle and intensity of sunlight can change throughout the day, leading to different levels of illumination and potential glare in morning videos. These variations in lighting conditions can affect the quality and reliability of visual features used by the tracker, resulting in decreased performance.

**Table 8:** Comparative results of finetuned trackers on D-PTUAC testing set per daytime.

	TATrack-Base384^26^	SeqTrack-B256^29^	SLT-TrDiMP^36^	MKDNet^17^	TrDiMP^35^	Super DiMP^32^	DropTrack^45^	ToMP50^22^	MixFormer-CvT^37^	AiATrack^34^
Evening	67.30	71.22**	67.31	63.92	74.39*	70.09***	67.24	69.99	63.94	67.30
Morning	63.06*	55.14	52.64	55.32	57.54	60.25***	52.30	57.93	52.04	61.63**

Trackers are ranked per SR (%) metric, with the best indicated by *, second best by **, and third best by ***

TrDiMP^24^ demonstrates the best performance in evening videos, surpassing the second best-performing tracker SeqTrack-B256^29^ by 4.45%. Moreover, the best-performing tracker in videos captured in the morning is TATrack-Base384^26^ with a performance improvement of 2.32% compared to the second best-performing tracker AiATrack^34^.

#### Attribute-wise performance

**Fig. 11:**
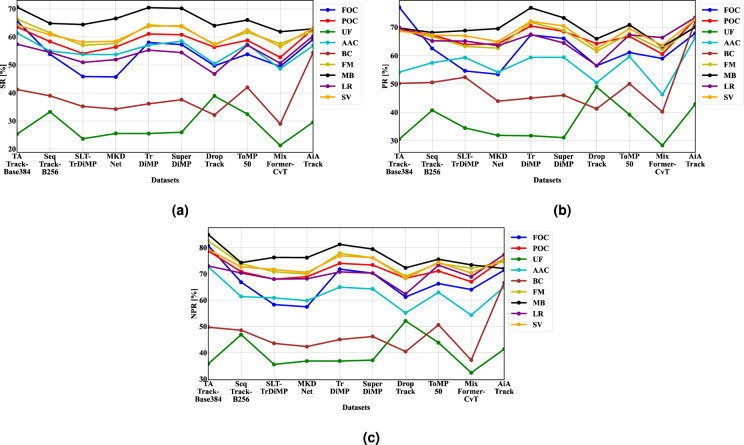
A visual comparative attribute-wise results of finetuned trackers for nine unique attributes on D-PTUAC testing set using, (**a**) SR (%), (**b**) PR (%), and (**c**) NPR (%). Please zoom for better clarity.

In order to comprehensively evaluate the performance of various tracking algorithms, we evaluated ten finetuned trackers on 17 attributes using the D-PTUAC testing set. The results of this evaluation are presented in Table 9. For better visualisation, we plot the top nine unique attributes in our D-PTUAC dataset against the performance of the chosen trackers as depicted in Fig. 11.

**Table 9:** Comparative attribute-wise results of finetuned trackers on D-PTUAC testing set.

Tracker	Metrics	UF	AAC	ARC	BC	DEF	IV	FM	LT	ST	LR	MB	OV	FOC	POC	PV	ROT	SV
TATrack-Base384^26^	SR (%)	25.34	61.35*	66.56*	41.23***	56.17***	64.01	66.36*	53.71**	70.78**	57.37**	70.59*	65.08	65.69*	63.56*	66.18*	62.25*	64.29*
	PR (%)	30.61	54.19	75.12*	50.19	69.00***	77.89***	68.79	59.64	75.32	69.95**	69.70	75.99	77.07*	69.27***	53.52	65.45	68.66
	NPR (%)	35.80	72.68*	84.33*	49.73***	70.13***	79.95	82.53*	66.47*	86.78*	73.02***	84.97*	86.15	80.75*	78.74*	78.87*	76.06*	79.31*
SeqTrack-B256^29^	SR (%)	33.28**	54.96	60.83	38.99	53.76	62.62	61.43	47.95	68.93	54.47	64.82	68.92**	53.81	58.33	59.26	57.71	60.79
	PR (%)	40.73***	57.46	70.38	50.55***	64.08	73.29	66.69	56.75	74.65	65.19	68.14	79.71***	62.52	67.31	58.34	63.65	67.26
	NPR (%)	46.89**	61.40	76.02	48.55	67.12	77.53	73.91	59.75	81.06	70.36	74.31	89.85**	66.87	70.87	65.47	68.29	72.65
SLT-TrDiMP^36^	SR (%)	23.66	53.76	57.72	35.21	50.82	58.81	57.01	48.09	64.13	50.91	64.40	64.03	45.86	54.08	57.71	55.87	58.09
	PR (%)	34.48	59.31	68.64	52.39**	64.61	72.49	63.26	59.04	72.44	65.14	68.82	76.09	54.62	63.88	58.09	64.58	66.98
	NPR (%)	35.55	60.90	75.02	43.60	65.66	75.99	70.84	60.63	78.62	68.04	76.28	87.69	58.31	68.03	64.77	68.12	71.72
MKDNet^17^	SR (%)	25.54	53.69	58.08	34.24	51.54	58.80	57.63	48.58	64.29	51.89	66.55	65.80	45.71	56.39	58.58	56.56	58.42
	PR (%)	31.91	54.17	67.79	43.95	62.32	70.47	62.63	55.36	71.13	63.50	69.50	78.43	53.41	64.14	55.69	62.18	64.92
	NPR (%)	36.85	59.83	73.29	42.34	64.64	74.17	69.99	60.81	76.62	68.19	76.20	88.44***	57.47	68.97	64.72	67.13	70.57
TrDiMP^35^	SR (%)	25.50	57.03	63.86**	36.15	57.26**	65.47**	64.28**	52.86***	70.43***	55.29	70.44**	67.44***	58.00***	61.06***	61.69***	61.89***	63.80***
	PR (%)	31.79	59.36	75.06**	45.07	69.19**	78.84*	71.76*	62.54**	78.84***	67.45***	76.86*	81.76**	67.30***	70.47**	61.51	69.46**	72.10**
	NPR (%)	36.86	64.99***	79.36**	45.08	70.59**	81.43**	77.87**	64.18	84.86***	70.78	81.24**	87.82	71.82**	74.05***	69.75**	73.39***	76.95**
SuperDiMP^32^	SR (%)	25.97	58.47**	63.39***	37.59	55.52	64.46***	63.64***	48.44	72.76*	54.42	70.16***	65.92	57.25	60.76	63.52**	61.10	64.00**
	PR (%)	31.11	59.40***	72.20***	46.02	65.72	75.46	68.89***	54.73	79.69*	64.48	73.31**	76.35	66.05	68.53	61.62***	66.83***	70.47***
	NPR (%)	37.18	64.31	78.18	46.21	69.28	80.16***	76.14***	59.80	85.52**	70.33	79.46***	86.34	70.29	73.41	68.90***	71.86	76.20***
DropTrack^45^	SR (%)	38.93*	50.45	57.98	32.14	51.28	60.47	57.48	43.13	66.48	46.78	64.01	59.37	49.71	56.34	53.69	55.15	57.13
	PR (%)	48.93*	50.45	66.11	41.27	61.31	71.11	61.52	51.68	70.74	56.51	65.93	69.50	56.44	64.22	49.74	60.12	62.54
	NPR (%)	52.12*	55.17	72.58	40.49	64.92	75.67	69.14	54.08	78.15	62.39	72.30	79.72	61.22	68.36	57.54	65.30	68.39
ToMP50^22^	SR (%)	32.46***	57.35***	63.30	41.99**	55.02	63.27	61.63	53.82*	67.57	57.08***	66.03	70.92*	53.80	58.78	61.56	59.17	62.44
	PR (%)	39.19	59.69**	72.15	50.06	65.22	74.06	67.76	61.00***	74.86	67.29	70.83***	82.53*	61.10	66.79	61.74**	65.73	69.49
	NPR (%)	43.84***	62.98	78.70***	50.61**	68.71	78.62	74.05	66.34**	79.29	73.34**	75.55	92.77*	66.29	71.12	66.86	69.78	74.40
MixFormer-CvT^37^	SR (%)	21.28	48.63	55.06	28.97	50.89	57.72	57.57	46.32	62.47	50.62	61.86	60.05	49.44	52.72	54.81	54.64	56.50
	PR (%)	28.35	46.29	66.91	40.21	65.77	72.50	62.17	56.14	68.68	66.36	63.21	75.89	58.96	60.53	47.70	61.29	63.56
	NPR (%)	32.37	54.33	72.69	37.23	66.56	75.88	72.16	60.25	76.57	68.91	73.44	84.58	64.04	67.02	60.59	66.74	70.36
AiATrack^34^	SR (%)	29.55	56.87	59.86	54.40*	63.42*	67.09*	61.99	52.41	70.16	60.35*	62.90	59.40	59.09**	62.76**	57.45	62.01**	63.14
	PR (%)	42.90**	66.37*	69.85	72.17*	76.19*	78.68**	70.32**	64.07*	79.01**	73.46*	70.02	68.92	67.88**	72.87*	63.80*	71.94*	72.76*
	NPR (%)	41.39	65.04**	74.78	66.66*	77.97*	82.26*	74.61	63.83***	83.31	77.33*	72.05	78.36	71.41***	75.77**	64.70	73.73**	75.67

Trackers are ranked in terms of SR (%), PR (%), and NPR (%) metrics per attribute, with the best indicated by*, second best by**, and third best by***.

TATrack-Base384^26^ and AiATrack^34^ demonstrate effective mitigation of challenges such as ACC, BC, FM, LT, ST, POC, FOC, PV, ROT, and SV when compared to other algorithms. It is worth highlighting that these challenges, particularly BC, UF, and OCC, significantly impact the quality of object features. The aforementioned trackers successfully address these challenges, demonstrating significant improvements in the representation, discrimination, and localization abilities for tracking multi-scale uniform appearance objects.

However, upon further analysis of the performance on attributes related to UF, BC, IV, and OV challenges, we observe that there remains a significant gap in effectively addressing challenges associated with tracking multi-scale uniform appearance objects. Consequently, further research and development efforts are necessary to overcome the challenges associated with tracking multi-scale uniform appearance objects in real-world applications.

#### Qualitative evaluation

**Fig. 12:**
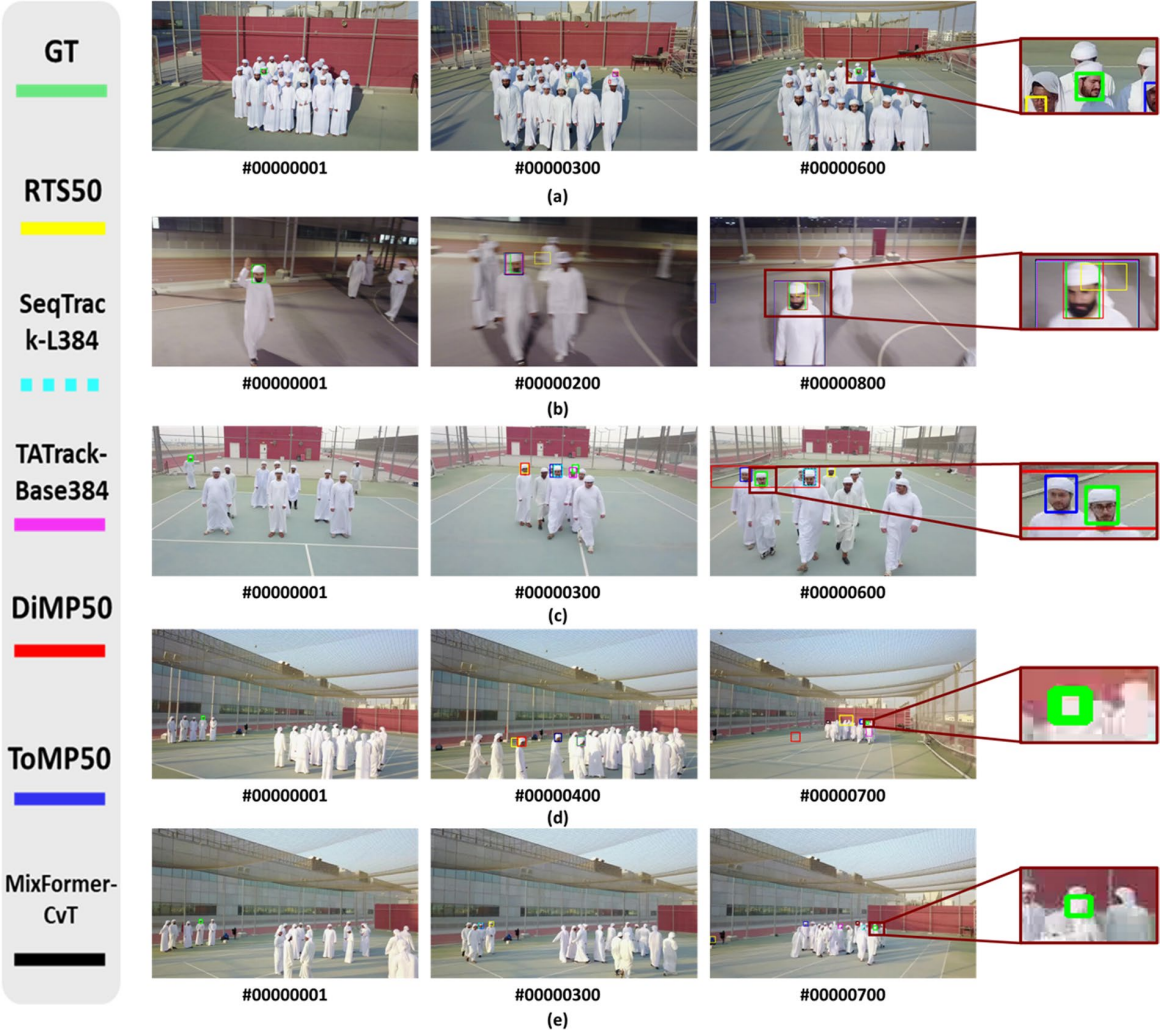
Qualitative evaluation on six representative trackers. To enhance visibility, we have magnified the object regions and presented them on the right side of the original images. The enlarged regions are shown for the following examples: (**a**) S1, (**b**) S2, (**c**) S3, (**d**) S4, and (**e**) S5.

For a qualitative assessment of different trackers and to provide insights for future research, we present the qualitative evaluation results in Fig. 12 of six representative trackers: RTS^28^, SeqTrackL-384^29^, TATrack-Base384^26^, DiMP50^24^, ToMP50^22^, and MixFormer-CvT^37^. We show in Fig. 12 five tracking scenarios with attributes such as AAC, ARC, SV, OCC, BC, LR, FM, MB, ROT, different CD levels, and different SS. Furthermore, to facilitate observation, we have enlarged the regions containing the target objects and presented them on the right side of the original images. In the D-PTUAC dataset, sequences often exhibit multiple challenge attributes, posing significant difficulties for tracking multi-scale uniform appearance objects and leading to frequent failures of current SOTA trackers. For instance, in Fig. 12a–c, in S1, S2, and S3 sequences present challenges such as ARC, FM, MB, OCC, SV, BC, LR, and UF, which pose considerable challenges for existing trackers.

Additionally, we present some examples of failed cases for SOTA trackers on the D-PTUAC dataset in Fig. 12d,e. These failed cases involve various challenge attributes, including AAC, ARC, SV, BC, MB, FM, OCC, BC, LR, and UF. The challenging attributes of FM can cause the target to move beyond the trackers’ search area. Although there are some re-detection tracking algorithms capable of addressing such problems, trackers often struggle to track the multi-scale uniform appearance object due to the lack of sufficient appearance information and interference from the BC. MB, often accompanied by FM and camera motion, further degrades the quality of feature representation. Moreover, as shown in Fig. 12d,e, the challenge attribute of OCC frequently results in model drift and targets moving beyond the search area. In summary, the main reasons for the failure of other trackers in tracking the D-PTUAC dataset can be attributed to (1) the LR, UF, and limited informative content of multi-scale uniform appearance objects, which hinder effective feature extraction and precise target localization, and (2) the presence of multiple challenge attributes within the same video sequence in the D-PTUAC dataset, posing substantial challenges for tracking methods.

## Usage Notes

The D-PTUAC dataset^19^, is publicly accessible on Figshare, available at (10.6084/m9.figshare.24590568.v2). This dataset is offered for unrestricted use, permitting users to freely copy, share, and distribute the data in any format or medium. Additionally, users are granted the flexibility to adapt, remix, transform, and build upon the material. In the pursuit of fostering reproducibility, the predicted bounding boxes and finetuned weights of the visual object trackers are also available on Figshare, accessible at (10.6084/m9.figshare.24590268.v2)^38^. Both the dataset and the evaluation scripts are licensed under the Creative Commons “Attribution 4.0 International” license, which can be reviewed at (https://creativecommons.org/licenses/by/4.0/) .

**Table 10:** Visual object trackers code availability.

Visual Object Tracker	URL
TATrack-Base384^26^	https://github.com/hekaijie123/TATrack
TATrack-Base^26^	
TATrack-Tiny^26^	
SeqTrack-B384^29^	https://github.com/microsoft/VideoX/tree/master/SeqTrack
SeqTrack-B256^29^	
SeqTrack-L384^29^	
SeqTrack-L256^29^	
SLT-TrDiMP^36^	https://github.com/byminji/SLTtrack
SLT-TrSiam^36^	
MKDNet^17^	https://github.com/ZYB0726/MKDNet
OSTrack256^41^	https://github.com/botaoye/OSTrack
OSTrack384^41^	
NeighborTrack-OSTrack384^41^	https://github.com/franktpmvu/NeighborTrack
AiATrack^34^	https://github.com/Little-Podi/AiATrack
TrDiMP^35^	https://github.com/594422814/TransformerTrack
TrSiam^35^	
DropTrack^45^	https://github.com/jimmy-dq/DropTrack
MixFormer-CvT^37^	https://github.com/MCG-NJU/MixFormer
MixFormer-CvT-L^37^	
MixFormer-ViT^37^	
MixFormer-ViT-L^37^	
MixFormer-ConvMAE^37^	
MixFormer-ConvMAE-L^37^	
ATOM^23^	https://github.com/visionml/pytracking
DiMP18^24^	
DiMP50^24^	
PrDiMP18^33^	
PrDiMP50^33^	
SuperDiMP^32^	
SuperDiMPSimple^32^	
ToMP50^22^	
ToMP101^22^	
KYS^40^	
KeepTrack^46^	
RTS^28^	
LwL^44^	
ECO^42^	
E.T.Track^43^	https://github.com/pblatter/ettrack
TrTr^30^	https://github.com/tongtybj/TrTr
TransT^31^	https://github.com/chenxin-dlut/TransT
STARK-ST101^25^	https://github.com/researchmm/Stark
STARK-ST50^25^	
STARK-S50^25^	
DeT-DiMP50-Mean^27^	https://github.com/xiaozai/DeT

## Data Availability

The transformation of RGB frames into estimated monocular depth frames was conducted using models from the works cited in^20,39^. These models are available at (https://github.com/isl-org/MiDaS). In a similar manner, the generation of segmentation masks from RGB frames was accomplished using the SAM model^21^, accessible from (https://github.com/facebookresearch/segment-anything). Additionally, the VOT algorithms employed in this study were sourced from the official codes provided by the respective authors, as detailed in Table 10. For ease of access and utilization, all relevant codes, finetuned models, and predicted bounding boxes of the visual object trackers have been collated on our project’s Figshare page. These resources can be accessed via the following links: (10.6084/m9.figshare.24590268.v2)^38^, and (https://github.com/HamadYA/D-PTUAC).
