# Author Correction: Drone-Person Tracking in Uniform Appearance Crowd: A New Dataset

**DOI:** 10.1038/s41597-025-04578-9

**Published:** 2025-02-18

**Authors:** Mohamad Alansari, Oussama Abdul Hay, Sara Alansari, Sajid Javed, Abdulhadi Shoufan, Yahya Zweiri, Naoufel Werghi

**Affiliations:** 1 Department of Computer Science, Khalifa University, Abu Dhabi, UAE; 2 Department of Aerospace Engineering, Khalifa University, Abu Dhabi, UAE; 3 Advanced Research and Innovation Center (ARIC), Khalifa University, Abu Dhabi, UAE; 4 Khalifa University Center for Autonomous Robotic Systems (KUCARS), Khalifa University, Abu Dhabi, UAE; 5 Center for Cyber-Physical Systems (C2PS), Khalifa University, Abu Dhabi, UAE

**Keywords:** Computer science, Computational science

Correction to: *Scientific Data* 10.1038/s41597-023-02810-y, published online 02 January 2024

In this article the affiliation details for all authors were missing ‘Khalifa University’. The original article has been corrected.

